# Proteome dataset of sea bass (*Dicentrarchus labrax*) skin-scales exposed to fluoxetine and estradiol

**DOI:** 10.1016/j.dib.2022.107971

**Published:** 2022-02-16

**Authors:** Liliana Anjos, Patrícia I.S. Pinto, Soraia Santos, M. Dulce Estêvão, Cátia Santa, Bruno Manadas, Adelino V.M. Canário, Deborah M. Power

**Affiliations:** a Centro de Ciências do Mar (CCMAR), Universidade do Algarve, 8005-139, Faro, Portugal; b Escola Superior de Saúde da Universidade do Algarve, Campus de Gambelas, 8005-139, Faro, Portugal; c CNC - Center for Neuroscience and Cell Biology, Universidade de Coimbra, 3004-517, Coimbra, Portugal; d Institute for Interdisciplinary Research (IIIUC), Universidade de Coimbra, 3004-517, Coimbra, Portugal; e Shanghai Ocean University International Center for Marine Studies, Shanghai 201306, China

**Keywords:** SWATH-MS quantitative proteomics, Scales, Emerging pollutants, Antidepressant, Estrogen

## Abstract

Contamination of aquatic ecosystems with anthropogenic pollutants, including pharmaceutical drugs, is a major concern worldwide. Aquatic organisms such as fish are particularly at risk of exposure to pollutants. The surface of fish is the first point of contact with pollutants, but few studies have considered the impact of pollutants on the skin-scale barrier. The present proteome data are the basis of the findings discussed in the associated research article “Proteomics of sea bass skin-scales exposed to the emerging pollutant fluoxetine compared to estradiol” [1]. Juvenile sea bass were exposed by intraperitoneal injections to: a) the antidepressant fluoxetine (FLX), a widely prescribed psychotropic drug and an emerging pollutant; b) the natural estrogen 17β-estradiol (E2) and c) the vehicle, coconut oil (control). The scale proteome of fish exposed to these compounds for 5 days was analysed using quantitative label-free proteomics technology SWATH-MS (sequential windowed data-independent acquisition of the total high-resolution-mass spectra). The proteome data generated was submitted to the ProteomeXchange Consortium via the PRIDE partner repository with the dataset identifier PXD020983. LC-MS data from pooled protein extracts from the scales of all experimental groups was acquired using information-dependent acquisition (IDA) and 1,254 proteins were identified by searching against the sea bass genome database. 715 proteins were quantified by SWATH acquisition, and 213 proteins had modified levels (p < 0.05) between the E2- or FLX-exposed fish compared to the control. The main biological processes and KEGG pathways affected by E2 or FLX treatments were identified using Cytoscape/ClueGO enrichment analyses.

## Specifications Table

**Table:** 

Subject	Molecular Biology
Specific subject area	Ecotoxicology, Endocrine disruption, Environment, Pollution.
Type of data	Table, figures, supplementary tables.
How data were acquired	Triple TOF 5600 (ABSciex) LC (nanoLC Ultra 2D, Eksigent)-SWATH-MS system with information-dependent acquisition (IDA).
Data format	Raw Analysed
Parameters for data collection	Marine cultured sea bass were exposed to estradiol and fluoxetine by intraperitoneal injection and 5 days later the scales and adhering epithelia were collected.
Description of data collection	Protein extraction, protein quantification and quality evaluation, short-GeLC-SWATH-MS proteome library preparation, protein identification and quantification, statistical analyses and bioinformatics analyses.
Data source location	Centro de Ciências do Mar (CCMAR), Faro, Algarve, Portugal (37° 1′ 0″ N; 7° 56′ 0″ W).
Data accessibility	Within the present article and deposited in the PRIDE repository (ProteomeXchange Consortium). Data identification number: PXD020983. Direct URL to data: http://www.ebi.ac.uk/pride/archive/projects/PXD020983
Related research article	P.I. Pinto, L. Anjos, M. Estêvão, S. Santos, C. Santa, B. Manadas, T. Monsinjon, A.V.M. Canário, D.M. Power, Proteomics of sea bass skin-scales exposed to the emerging pollutant fluoxetine compared to estradiol, Science of The Total Environment (2021) 152671 [1]. https://doi.org/10.1016/j.scitotenv.2021.152671

## Value of the Data

• This is a comprehensive study of the proteome of fish scales and how it is affected by fluoxetine (FLX, an emerging pollutant) and estradiol (E2, a natural hormone and representative of diverse estrogenic endocrine-disrupting pollutants). The study provides insight into the function of estrogens in scale physiology and the potential impacts of exposure to FLX and estrogenic pollutants.
• The dataset is relevant for aquaculture, environmental protection and ecotoxicology, since anthropogenic compounds can modify marine vertebrate physiology through their effects on the endocrine system.
• SWATH-MS quantitative proteomics gave insight into the proteome of fish scales and adhering epithelia and how they changed when fish were exposed to estradiol and fluoxetine. The proteins with different levels are relevant for studies directed at understanding the likely impact of environmental pollutants fluoxetine and estradiol on fish physiology.
• The proteins responsive to estradiol and fluoxetine provide the basis for potential biomarker development to assess environmental pollution and chemical risk assessment.

## 1 Data Description

Table 1 summarizes the number of proteins and peptides detected in the sea bass scale protein extracts that were identified by information-dependent acquisition (IDA) or quantified by SWATH analysis in sea bass treated with: i) estradiol (E2), ii) fluoxetine (FLX) or iii) coconut oil only (C - control). This table shows the number of proteins/peptides identified for each of the two individual libraries (“pooled C_library” - prepared from pools of control samples only; and “pooled C, E2, FLX_library”- prepared by combining all the IDA files obtained for the 3 independent pools of each experimental condition) and the number of proteins confidently quantified by SWATH (local FDR < 0.05, p < 0.05), including proteins with a modified level in the treatment groups compared to control scales.

**Table 1:** **The number of proteins and peptides in the SWATH proteomic analysis obtained from the sea bass protein extracts of scales from treated and/or control animals.** The number of identified proteins/peptides is shown for each of the two individual libraries generated (IDA analysis): “Pooled C_library” (prepared from pools of control samples) and for the global identification library “Pooled C, E2, FLX_library”, that was generated by combining all the files obtained from the 3 independent pools of each experimental condition and was used as the library for SWATH quantification. The number of identified/quantified proteins obtained by SWATH processing under confident quantitative values (local FDR < 0.05, p < 0.05) and the number of proteins with modified levels due to E2 of FLX treatments (p < 0.05) are presented, as well as the corresponding Tables where detailed results can be found.

	N° of Proteins	N° of Peptides	Supported by ≥ 3 peptides (95%)	Table link
***(IDA) Identification - Library_experimental group***				

Pooled C_library	985	5710	560	Supp. Table 1_worksheet-ProteinSummary
Pooled C, E2, FLX_library	1254	8073	728	Supp. Table 3_worksheet-ProteinSummary

***SWATH quantification***				

**Total identified and quantified**	715	−	−	Supp. Table 4_worksheet-Proteins_ITotal
**Total modified**	213	−	−	Supp. Table 4 and Table S4 in the research manuscript [1]
**Total modified by E2**	110	−	−	Supp. Table 4 and Table S4 in the research manuscript [1]
Up	89	−	−	Supp. Table 4 and Table S4 in the research manuscript [1]
Down	21	−	−	Supp. Table 4 and Table S4 in the research manuscript [1]
**Total modified by FLX**	134	−	−	Supp. Table 4 and Table S4 in the research manuscript [1]
Up	55	−	−	Supp. Table 4 and Table S4 in the research manuscript [1]
Down	79	−	−	Supp. Table 4 and Table S4 in the research manuscript [1]
**E2, FLX common modified proteins**	31	−	−	Supp. Table 4 and Table S4 in the research manuscript [1]
**Unique modified proteins by E2**	79	−	−	Supp. Table 4 and Table S4 in the research manuscript [1]
**Unique modified proteins by FLX**	103	−	−	Supp. Table 4 and Table S4 in the research manuscript [1]

**Figure 1:**
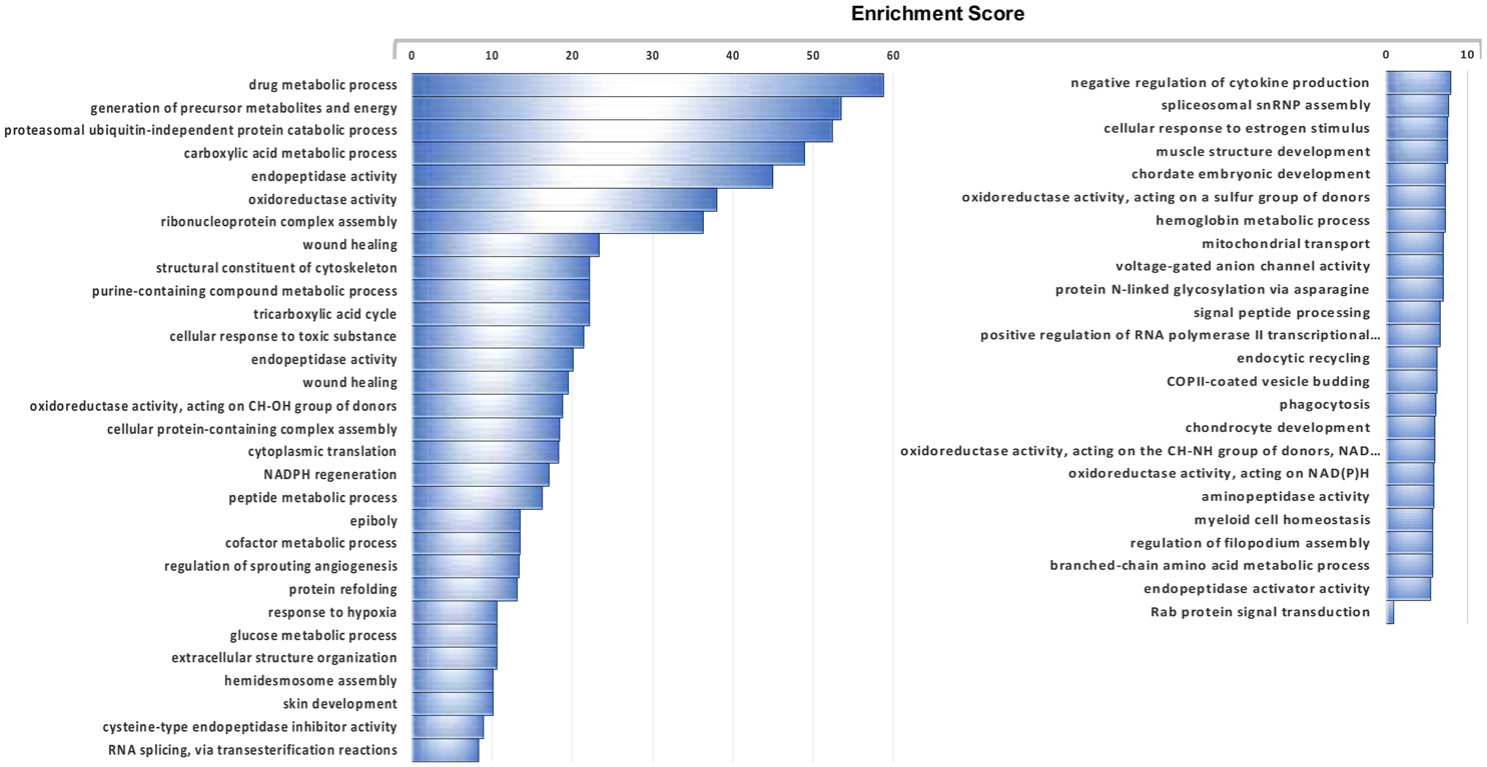
**Functional enrichment analyses of all proteins identified in protein extracts of sea bass scales (control samples).** The enriched Gene Ontology Biological Processes (GO-BP) that were overrepresented in the global sea bass scale proteome are identified (see **Suppl. Table 1** for the list of identified proteins). Enrichment for GO terms was carried out using the ClueGO plugin and Cytoscape software, which found 562 terms with significant enrichment (FDR < 0.05). Represented are 55 functional groups with significant enrichment (FDR  <  0.05) that clustered the 562 significant GO terms according to their functional classification. For detailed lists of all significantly enriched GO terms and groups and associated KEGG pathways consult **Suppl. Table 2**. The length of bars represents the significance of each group in the ClueGO network output measured by the enrichment score (−log2 (group FDR)).

**Figure 2:**
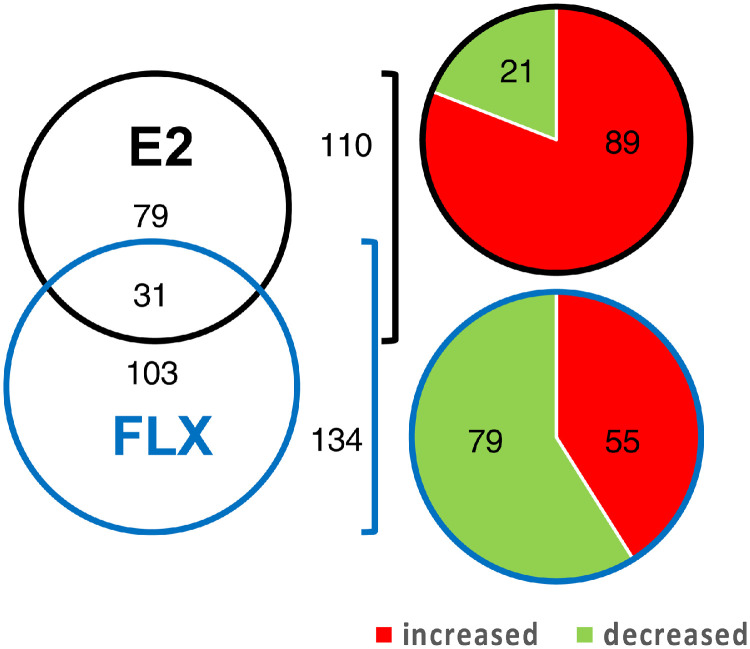
**Number of proteins in the sea bass scale proteome with a differential expression in response to estradiol or fluoxetine exposure.** The Venn diagram shows the number of proteins with significantly different levels between the control scales and scales exposed to estradiol (E2) or to fluoxetine (FLX) for 5 days (FDR < 0.05). The pie charts correspond to the total number of proteins affected by E2 (110) or by FLX (134). The percentage of proteins that increased are shown in red and the percentage of proteins that decreased are shown in green. The size of the circles in the Venn diagram and pie charts is proportional to the number of proteins with modified expression in each treatment compared to the control.

Supplementary Table 1 presents the IDA identification results obtained for the protein extracts from the control sea bass scales, and lists the proteins identified using the predicted proteins in the sea bass genome and corresponds to the “Pooled C_library”. Fig. 1. presents the functional enrichment analyses derived from the enriched Gene Ontology Biological Processes (GO-BP) that were overrepresented in the control sea bass scale proteome (using the proteins identified in the “Pooled C_library”). The detailed lists of the overrepresented GO-BP terms in the control scales as well as overrepresented KEGG pathways are presented in Supplementary Table 2. Supplementary Table 3 presents the library of identified proteins obtained from the files of the 3 independent pools corresponding to each experimental condition “Pooled C, E2, FLX_library”. All the proteins and peptides in sea bass protein extracts of scales from the three experimental groups that were identified by IDA analysis (using the predicted proteins from the sea bass genome) are listed. Supplementary Table 4 lists the quantification parameters of the total proteome of sea bass scales for each of the seven biological replicates per treatment group (C, E2 or FLX) obtained by SWATH processing of the 3 independent experimental groups. The differential proteins identified in the ProteinsITotal worksheet correspond to proteins with significantly altered levels between the treatment groups and the control group when the threshold for significant change was set at p < 0.05. Fig. 2 shows a Venn diagram comparing the number of differentially expressed proteins in E2 or FLX treatments, as well as commonly affected proteins. The pie charts present the percentage of the identified proteins in sea bass scales that increased or decreased in response to treatments. Supplementary Table 5 presents significantly enriched GO-BP and KEGG pathways identified using the proteins that were significantly modified in scales from animals exposed to E2 and/or FLX (analysis “All”). Supplementary Table 6 and 7 present the significantly enriched GO-BP and KEGGs (FDR < 0.05) in the sub-sets of proteins that had an increased expression in scales from fish exposed to E2 and had a decreased expression in scales from fish exposed to FLX, respectively. Supplementary Tables 8 and 9 list the significantly enriched KEGG (FDR < 0.05) pathways found for the proteins with decreased levels in scale extracts from E2 exposed animals or increased levels in scale extracts from FLX exposed animals, respectively, while no significantly enriched GO-BPs were observed for the same lists of proteins.

## 2 Experimental Design, Materials and Methods

SWATH-MS (sequential window data-independent acquisition of the total high-resolution-mass spectra) quantitative proteomic analysis was used to generate a global proteome of sea bass (*Dicentrarchus labrax*) scales with adhering epithelial cells (designated scales from now on). In addition, the change in the proteome of scales from fish exposed to estradiol or to fluoxetine 5 days after injection was characterized.

### 2.1 Experimental setup and sample collection

Animal maintenance and experimentation followed national (DL 113/2013) and international (EU Directive 2010/63/UE) guidelines. Full details of the experimental design and sampling can be found in the associated research paper [1]. Briefly, to assess the effect of estradiol (E2) or fluoxetine (FLX) on the scale proteome of sea bass*,* three experimental groups were established with replicate tanks for each experimental group (with n = 5 fish per replicate tank per group). The collected tissue was designated “scales” and was composed of scales and their adhering epithelial cells, which were not dissociated during sampling. Sampling of scales was done by plucking them from the skin using forceps, in a standardized way for all fish and treatments. The fish in the treatment groups received a single intraperitoneal (i.p.) injection of coconut oil (as described in Pinto [2]) containing E2 (Sigma-Aldrich) or FLX hydrochloride (Cayman) at 5 mg/kg body weight each treatment or in the case of the control group, coconut oil alone (vehicle, 3 ml/kg body weight). After 5 days of exposure to the chemicals, the scales localized below the dorsal fin were collected from all the fish in each experimental group (n = 10/group) using forceps and immediately frozen in liquid nitrogen and stored at -80 °C until protein extraction.

### 2.2 Plasma and scale analyses

The methods used for analysis of plasma and scales have previously been described in Pinto [2]. In order to confirm the effectiveness of the treatments, estradiol was quantified by radioimmunoassay (RIA) in duplicate heat denatured plasma samples of each individual from all experimental groups (n = 9 - 10/group) as previously described [2].

Plasma levels of FLX and its metabolite norfluoxetine (nor-FLX) were measured by specific LC-MS using QuEChERS (quick easy cheap effective rugged safe) extracts of individual plasma samples (n = 9 - 10/group) using the method described by Robert [3], optimized for fish plasma as detailed below. 80 µL of plasma was placed into an ice-cold microvial and lyophilized using a Bioblock Scientific alpha 2 - 4 LD lyophilizer prior to homogenization. The following extraction buffers were added to the plasma together with ceramic beads in consecutive steps: (i) 400 µL of phosphate buffer (0.1 M, pH 10.5); (ii) 150 mg of Bond Elute QuEChERS AOAC (Agilent, USA); (iii) 10 µl of deuterated (d5)- FLX hydrochloride (Sigma-Aldrich, USA) at 0.01 ng/ml and (iv) 320 µL of acetonitrile, followed by homogenisation for 30 s each using a Precellys tissue homogeniser (Bertin, France). After centrifugation at 8000 x g, for 5 min at 10 °C, the supernatant was transferred to a clean microvial containing 60 mg of dispersed solid-phase extraction solution (Agilent). Samples were vigorously shaken, centrifuged at 8000 x g for 5 min at 10 °C and the supernatant was transferred to low absorption vials for subsequent LC-MS analysis. Deuterated FLX and nor-FLX were used as internal standards, using stock solutions prepared in 65:35 (v/v) H_2_O/acetonitrile in a final concentration of 100 ng/mL. Calibration curve solutions (with concentrations varying between 0.49 and 5000 ng/mL prepared in water) were also spiked with the internal standards and submitted to the sample extraction procedure. Deuterated internal standards were monitored throughout the extraction and quantification processes to account for possible experimental errors or losses and high recovery rates were obtained (80 - 100 %). Separations were carried out on an Agilent 1200 liquid chromatography (LC) system using a Poroshell EC-C18 column (2.7 µm, 50 × 2.1 mm I.D.) at 25 °C followed by tandem mass spectrometry (MS/MS) performed on an Agilent 6340 Ion trap LC/MS, as previously described [3]. FLX and nor-FLX results were expressed in ng of compound/mL of plasma and reflect the automatic calibration run for all samples by the Agilent software based on the deuterated internal standards.

To measure the impact of E2/FLX exposure on biomarkers of mineral homeostasis in all experimental groups (detailed in the associated research paper [1]), enzymatic activities were measured as in Pinto [5] and scale mineral content was quantified as in Pinto [4], with modifications in the initial digestion to allow quantification of the phosphorus (P) content in addition to calcium, magnesium (Mg) and sodium (Na). In brief, 10 - 30 scales per fish were defrosted, washed 3 times in milli-Q water and dried at 105 °C for 3 h, after which their dry weight (5 - 22 mg) was recorded. Scales from each fish were digested with 5 ml of concentrated nitric acid (≥ 69.0 %, TraceSELECT™ for trace analysis, Fluka, USA) in an automated microwave digestion system (CEM Discover SP-D 80) using Synergy™ application software (CEM Corporation), for 10 min at 200 °C, 106 PSI and under agitation. Minerals were quantified in an Agilent microwave plasma-atomic emission spectrometer (MP-AES) model 4200, using the following dilutions of scale samples: 1:13.2 (to reach a concentration of 5 % nitric acid in all samples and standard curves) for Mg (measured at 285.2 nm) and Na (589.6 nm), and dilutions ranging from 1:13.2 to 1:264 in 5 % nitric acid for Ca (393.4 nm) and P (213.6 nm). The same MP-AES running parameters optimized in Pinto [4] were used and the standard curves ranged from 0.25 to 10 parts per million (ppm). Results in ppm were converted to µmol of each mineral and normalized by the scale dry weight for each sample.

### 2.3 Scale quantitative proteomic analysis

#### 2.3.1 Scales protein extracts

Total protein was extracted from the previously collected sea bass frozen scales for SWATH-MS quantitative proteomics analysis. The methodology used was a modification of the method previously reported in Anjos and Tsironi [6,7]. In brief, frozen scales (30 - 60 mg of scales per fish) were resuspended in 1 ml of ice-cold protein extraction buffer (50 mM Tris buffer at pH 6.8, 100 mM DTT, 1.7 % SDS) in 13 ml plastic tubes (SARSTEDT; Germany). Samples were homogenised by mechanical disruption using an Ultra Turrax homogeniser (IKA, Germany) equipped with a dispersing element S25N-8G-ST (for fibrous tissues). Homogenization was carried out at the maximum speed, using five cycles of 10 seconds with incubation on ice between cycles. To increase protein solubilisation, the homogenates were boiled at 95 °C for 10 min in a dry bath and allowed to cool at room temperature. Soluble protein fractions were separated by centrifugation, quantified, and submitted to quality control analysis by visual inspection of their electrophoretic profiles. These were obtained by fractionation of 10 µg of each total extract on 1D SDS-PAGE (10 %) using the Laemmli method [8], followed by staining with a Coomassie blue R solution (40 % v/v methanol; 10 % v/v acetic acid; 0.1 % w/v Coomassie blue R 250, Sigma–Aldrich, USA). All the protein extracts from the ten independent biological replicates per treatment group presented good quality and quantity. Seven replicates were chosen for subsequent quantitative proteomic analysis, based on the similarity between fish biometric (weight and length) parameters and on the plasma and scale measurements (detailed in the associated research paper [1]).

#### 2.3.2 Gel digestion and peptide extraction

Soluble protein from scale extracts were subjected to in-gel digestion following the short-GeLC method previously described [9]. In brief, the protein content of the scale extracts was assessed using a 2-D Quant Kit (GE Healthcare, USA) and 100 µg of each individual replicate sample per group and 3 pooled protein extracts from the replicates in each group (7 × 20 µg, Pool Control, Pool E2 and Pool FLX), were mixed with glycerol, bromophenol blue and a recombinant protein (Green fluorescent protein and Maltose-binding periplasmic protein (malE-GFP) that served as the internal standard). Thermally denatured protein samples (5 min at 95 °C) were partially fractionated on a precast gel (4-20 % Mini-Protean® TGX^TM^ Gel, Bio-Rad) for 15 min at 110 V. Proteins were subsequently stained with Colloidal Coomassie Blue, then destained and the gel regions containing protein were excised (3 fractions) and processed (destaining to in-gel protein digestion with trypsin, peptide extraction and solid-phase extraction with C18 sorbent (OMIX tip, Agilent Technologies) as reported in Santa [10]. The peptides isolated by solid-phase extraction were resuspended in the mobile phase used for liquid chromatography, 2 % acetonitrile (ACN) in 0.1 % formic acid (FA).

#### 2.3.3 LC-SWATH-MS acquisition

Samples were analysed on a Triple TOF™ 5600 System (Sciex®, Framingham, MA) through two operative phases: Information-dependent acquisition (IDA) of the pooled samples (Pool control (C), Pool E2 and Pool FLX); and the subsequent SWATH acquisition of each replicate sample from the 3 experimental conditions (n = 7 individual replicates per experimental condition). Peptide separation was obtained by liquid chromatography (nanoLC Ultra 2D, Eksigent®, CA) and IDA experiments were performed following the methodology described in Anjos [6] with the exception that the LC linear gradient was 5 % to 30 % ACN in 0.1 % FA and the mass spectrometer was operated with Analyst® TF 1.6, ABSciex®. The same chromatographic conditions used for IDA acquisitions were maintained for SWATH-MS analysis. The SWATH setup was based on Gillet [11] using the same parameters described in Anjos [6] with the exception that the survey scan acquired at the beginning of each cycle for instrument calibration was of 250 ms in between the 350 - 1500 m/z precursor range.

#### 2.3.4 Protein library generation and SWATH data processing

Two specific spectral libraries of precursor masses and fragment ions were created by combining all the MS/MS spectra generated from the pooled peptide mixtures in the IDA experiments, the “Pooled C_library” and the “Pooled C, E2, FLX_library”, with the latter used for subsequent SWATH processing after combining all the files obtained from the 3 independent pools. Peptide identification and library generation were carried out using Protein Pilot software (v5.0, ABSciex®). Search parameters used were: i) comparison against the predicted proteins from the sea bass genome database (assembly dicLab v1.0c with annotation from July 2013 and protein prediction from May 2015; file diclab1_pep.faa.gz downloaded from http://seabass.mpipz.mpg.de/DOWNLOADS/ in March 2016, [12]) and using ii) acrylamide alkylated cysteines as the fixed modification; iii) trypsin as digestion type (Paragon^TM^ Algorithm). An independent False Discovery Rate (FDR) analysis, using the target-decoy approach provided by Protein Pilot software, was used to assess the quality of protein identifications and positive identifications of proteins and peptides was accepted when local FDR reached 5 %. Data processing was carried out using the SWATH^TM^ processing plug-in for PeakView^TM^ (v2.0.01, ABSciex®) as described in Anjo [9] with minor modifications. Peptides were selected automatically from the C, E2, FLX pooled library as described in Anjos [6] and selection criteria included: i) unique peptides for a specific targeted protein were ranked by the intensity of the precursor ion from the IDA analysis; ii) peptides that contained biological modifications and/or were shared between different protein entries/isoforms were excluded. After retention time adjustment using the internal standard *mal*E-GFP peptides, up to 15 peptides, with up to 5 fragments each, were automatically selected per protein, and quantification was attempted for all proteins from the library that had a score in Protein Pilot below 5 % local FDR. The peak group confidence threshold was determined based on an FDR analysis using the target-decoy approach and 1 % extraction FDR threshold was used for all the analyses. Peptides that met the 1 % FDR threshold in at least three of the 7 biological replicates were retained, and the peak areas of the target fragment ions of those peptides were extracted across the experiments using an extracted-ion chromatogram (XIC) window of 3 min. The levels of the proteins were estimated by summing all the transitions from all the peptides for a given protein as described in Collins [13] and normalized to the total intensity of the sample. The mass spectrometry proteomics data have been deposited in the PRIDE partner repository of the ProteomeXchange Consortium [14,15] and has the dataset identifier PXD020983.

To compare the relative protein abundance in the proteomic data from E2 or FLX treatments the fold-change in protein level was calculated based on the medians between the treatments and the control groups. Statistical analysis was performed in SPSS (version 2.3, IBM) software using a Mann-Whitney (MW) pairwise test for comparison between treated and control groups. A p-value < 0.05 was considered statistically significant.

#### 2.3.5 Bioinformatic analysis

The zebrafish (*Danio rerio*) orthologues for all the sea bass proteins were obtained using stand-alone BlastX (with E value < 10^-10^) against the Ensembl zebrafish protein predictions (GRC Zebrafish Build 10, INSDC Assembly GCA_000002035.3 assessed at https://www. ensembl.org in September 2018, [16]). Protein lists that changed with the experimental conditions were analysed using the proportional Venn diagram tool BioVenn (http://www.biovenn.nl) [17], and the corresponding protein annotation and symbol was manually verified through: 1) BlastP against UniProtKB/Swiss-Prot (https://www.uniprot.org/) [18], Ensembl zebrafish and NCBI (https://www.ncbi.nlm.nih.gov/protein/) databases; and also 2) searches in GeneCards V.4.14 (https://www.genecards.org, [19]) and HGNC (https://www.genenames.org) databases. Functional analyses of gene ontology biological processes (GO-BP) and pathway (Kyoto Encyclopaedia of Genes and Genomes-KEGG) enrichment were run with the Cytoscape-ClueGO plug-in [20] using as the input zebrafish orthologues for each protein identified in sea bass, after removing duplicate zebrafish accessions. Enrichment analyses (using a right-sided hypergeometric test) were run selecting the GO-BP terms for zebrafish between levels 3 – 8. Terms were considered significantly enriched at an FDR (Benjamini–Hochberg) p ≤  0.05 and a minimum of 3 genes/4 % of the GO-BP proteins represented in the list. Enriched GO terms were grouped into functionally related networks using an initial group size of 1, a group merging setting of 50 % and a Kappa score of 0.4. The same parameters and strategy as described for GO-BP analyses were used for the parallel KEGG pathways enrichment analyses.

## Ethics Statement

Animal maintenance and experimentation followed national (DL 113/2013) and international (EU Directive 2010/63/UE) guidelines.

## CRediT Author Statement

**Liliana Anjos:** methodology, investigation, data curation, writing-original draft, review and editing, and visualization and funding acquisition; **Patricia Pinto:** conceptualization, methodology, investigation, formal analysis, data curation, writing-original draft, review and editing, visualization, project administration and funding acquisition; **Soraia Santos:** investigation, formal analysis, writing-review. **M. Dulce Estêvão:** formal analysis, writing-original draft, review and editing, and visualization; **Cátia Santa:** methodology, investigation, formal analysis, data curation and writing–review; **Bruno Manadas:** resources, methodology and writing–review; **Adelino Canário:** resources, supervision, funding acquisition and writing–review; **Deborah Power:** conceptualization, resources, writing-original draft, review and editing, supervision, project administration and funding acquisition.

## Associated Research Article

Patricia I. Pinto, L. Anjos, M. D. Estêvão, S. Santos, C. Santa, B. Manadas, T. Monsinjon, Adelino V.M. Canário, D.M. Power. “Proteomics of sea bass skin-scales exposed to the emerging pollutant fluoxetine compared to estradiol”, co-submitted to Science of the Total Environment [1].

## Declaration of competing Interest

The authors declare that they have no known competing financial interests or personal relationships which have, or could be perceived to have, influenced the work reported in this article.

## Data Availability

Title Dataset of the proteome profiles of sea bass (Dicentrarchus labrax) skin-scale exposed to estradiol and fluoxetine (Original data) (PRIDE).

## Appendix Supplementary materials

Image, application 1
