# Supplementary material for: Proteome dataset of sea bass (Dicentrarchus labrax) skin-scales exposed to fluoxetine and estradiol
Source: Data Brief. 2022 Feb 16;41:107971. doi: 10.1016/j.dib.2022.107971 (PMC8889360; doi:10.1016/j.dib.2022.107971)
Supplement: Supplementary file 1 [file mmc1.zip › 1_DIB (2)/Table 1- Balance ProtPep_August2021.docx]

**Table 1** - T**he number of proteins and peptides in the SWATH proteomic analysis obtained from the sea bass protein extracts of scales from treated and/or control animals.** The number of identified proteins/peptides is shown for each of the two individual libraries generated (IDA analysis): “pooled C_library” (prepared from pools of control samples) and for the global identification library “pooled C, E2, FLX_library”, that was generated by combining all the files obtained from the 3 independent pools of each experimental condition and was used as the library for SWATH quantification. The number of identified/quantified proteins obtained by SWATH processing under confident quantitative values (local FDR <0.05, p<0.05) and the number of proteins with modified levels due to E2 of FLX treatments (p<0.05) are presented, as well as the corresponding Tables where detailed results can be found.

|  | **Nº of Proteins** | **Nº of Peptides** | **Suported by ≥3 peptides (95%)** | **Table link** |
| --- | --- | --- | --- | --- |
| ***(IDA) Identification - Library_experimental group*** | | | | |
| Pooled C_library | 985 | 5710 | 560 | Supp. Table 1_worksheet-ProteinSummary |
| Pooled C, E2, FLX_library | 1254 | 8073 | 728 | Supp. Table 3_worksheet-ProteinSummary |
| ***SWATH quantification*** | | | | |
| **Total identified and quantified** | 715 | − | − | Supp. Table 4_worksheet-Proteins_ITotal |
| **Total modified** | 213 | − | − | Supp. Table 4 and Table S4 in the research manuscript [1] |
| **Total modified by E2** | 110 | − | − | Supp. Table 4 and Table S4 in the research manuscript [1] |
| Up | 89 | − | − | Supp. Table 4 and Table S4 in the research manuscript [1] |
| Down | 21 | − | − | Supp. Table 4 and Table S4 in the research manuscript [1] |
| **Total modified by FLX** | 134 | − | − | Supp. Table 4 and Table S4 in the research manuscript [1] |
| Up | 55 | − | − | Supp. Table 4 and Table S4 in the research manuscript [1] |
| Down | 79 | − | − | Supp. Table 4 and Table S4 in the research manuscript [1] |
| **E2, FLX modified in common** | 31 | − | − | Supp. Table 4 and Table S4 in the research manuscript [1] |
| **Unique modified by E2** | 79 | − | − | Supp. Table 4 and Table S4 in the research manuscript [1] |
| **Unique modified by FLX** | 103 | − | − | Supp. Table 4 and Table S4 in the research manuscript [1] |
