# Supplementary figures and images for: Proteome dataset of sea bass (Dicentrarchus labrax) skin-scales exposed to fluoxetine and estradiol
Source: Data Brief. 2022 Feb 16;41:107971. doi: 10.1016/j.dib.2022.107971 (PMC8889360; doi:10.1016/j.dib.2022.107971)

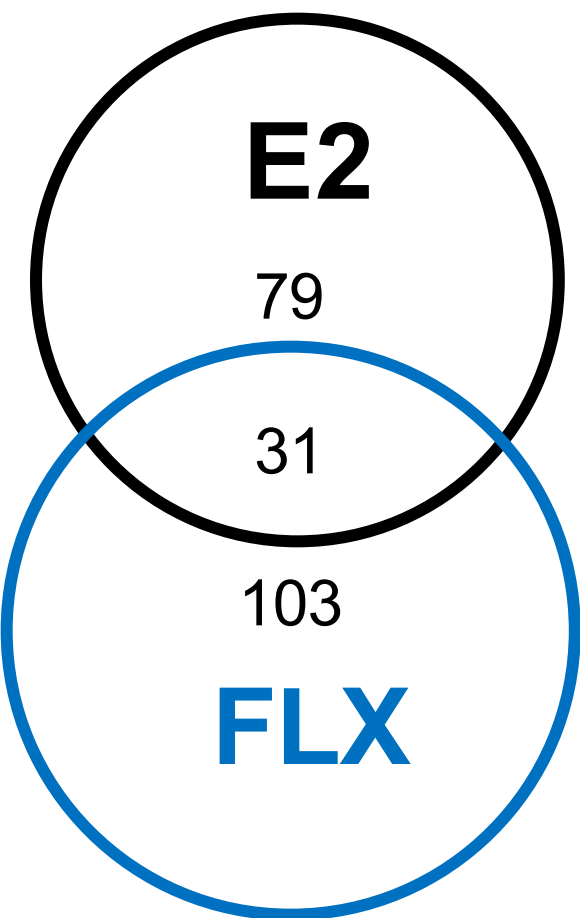

110

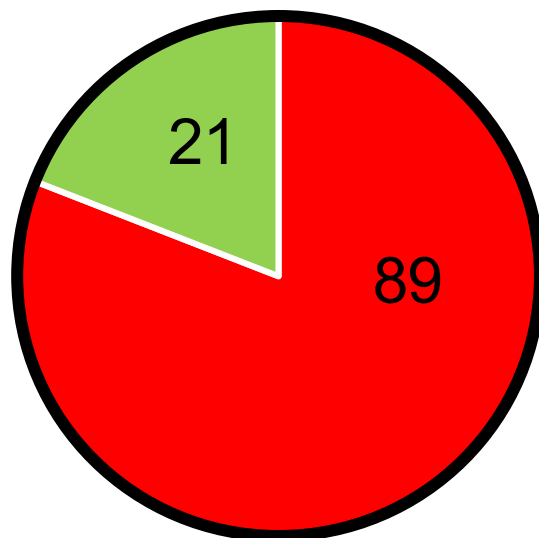

134

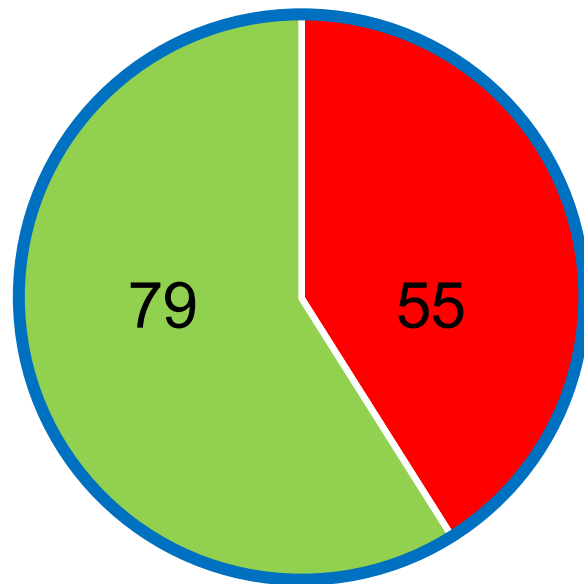

■ increased ■ decreased

Supplement: Supplementary file 1 [file mmc1.zip › 1_DIB (2)/Fig 2.pdf]

# Enrichment Score

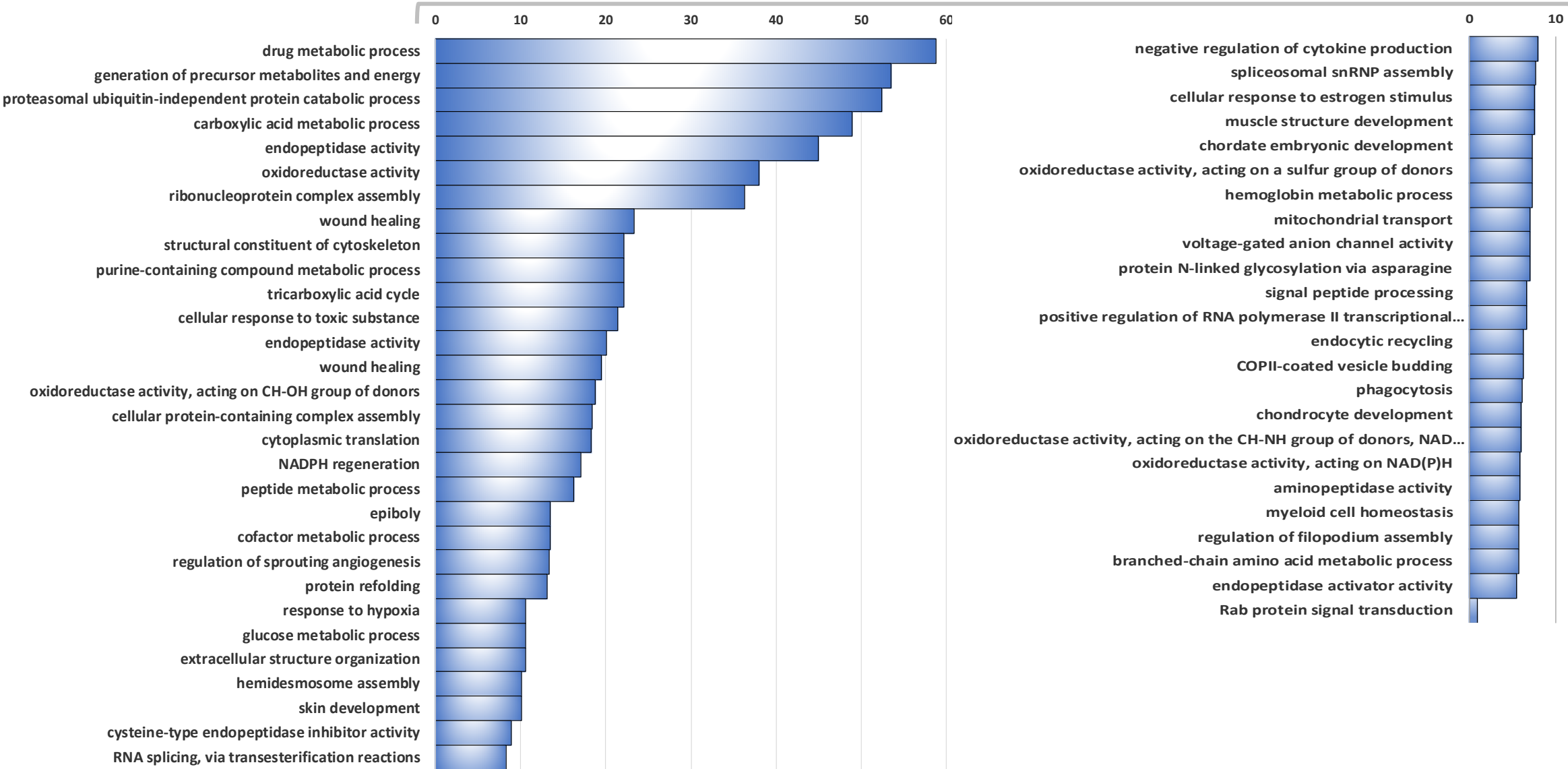

Supplement: Supplementary file 1 [file mmc1.zip › 1_DIB (2)/Fig 1.pdf]
